# A Nonsense Variant in the *ACADVL* Gene in German Hunting Terriers with Exercise Induced Metabolic Myopathy

**DOI:** 10.1534/g3.118.200084

**Published:** 2018-02-28

**Authors:** Vincent Lepori, Franziska Mühlhause, Adrian C. Sewell, Vidhya Jagannathan, Nils Janzen, Marco Rosati, Filipe Miguel Maximiano Alves de Sousa, Aurélie Tschopp, Gertraud Schüpbach, Kaspar Matiasek, Andrea Tipold, Tosso Leeb, Marion Kornberg

**Affiliations:** * Institute of Genetics, Vetsuisse Faculty, University of Bern, 3001, Switzerland; † Veterinary Clinic for Small Animals Elmer-Kornberg-Schanen, 54294 Trier, Germany; ‡ Department of Small Animal Medicine and Surgery, University of Veterinary Medicine Hannover, 30559, Germany; § Biocontrol, Laboratory for Veterinary Diagnostics, 55218 Ingelheim, Germany; ** Screening-Labor Hannover, 30430, Germany; †† Department of Clinical Chemistry, Hannover Medical School, 30625, Germany; ‡‡ Section of Clinical & Comparative Neuropathology, Institute of Veterinary Pathology, Centre for Clinical Veterinary Medicine, Ludwig-Maximilians-University, 80539 Munich, Germany; §§ Veterinary Public Health Institute, Vetsuisse Faculty, University of Bern, 3097 Liebefeld, Switzerland

**Keywords:** dog, canis lupus familiaris, metabolism, myopathy, beta-oxidation, very long-chain acyl-CoA dehydrogenase deficiency, whole genome sequencing, animal model

## Abstract

Several enzymes are involved in fatty acid oxidation, which is a key process in mitochondrial energy production. Inherited defects affecting any step of fatty acid oxidation can result in clinical disease. We present here an extended family of German Hunting Terriers with 10 dogs affected by clinical signs of exercise induced weakness, muscle pain, and suspected rhabdomyolysis. The combination of clinical signs, muscle histopathology and acylcarnitine analysis with an elevated tetradecenoylcarnitine (C14:1) peak suggested a possible diagnosis of acyl-CoA dehydrogenase very long chain deficiency (ACADVLD). Whole genome sequence analysis of one affected dog and 191 controls revealed a nonsense variant in the *ACADVL* gene encoding acyl-CoA dehydrogenase very long chain, c.1728C>A or p.(Tyr576*). The variant showed perfect association with the phenotype in the 10 affected and more than 500 control dogs of various breeds. Pathogenic variants in the *ACADVL* gene have been reported in humans with similar myopathic phenotypes. We therefore considered the detected variant to be the most likely candidate causative variant for the observed exercise induced myopathy. To our knowledge, this is the first description of this disease in dogs, which we propose to name exercise induced metabolic myopathy (EIMM), and the identification of the first canine pathogenic *ACADVL* variant. Our findings provide a large animal model for a known human disease and will enable genetic testing to avoid the unintentional breeding of affected offspring.

Mitochondrial β-oxidation of fatty acids is one of the major sources of cellular energy, in particular in organs with a high metabolic rate such as heart, liver and skeletal muscle. During fasting or prolonged exercise, ketone bodies produced in the liver become the major source to meet energy requirements (Andresen *et al.* 1999; Bartlett and Eaton 2004; Hisahara *et al.* 2015) whereby long-chain fatty acids are released from triglycerides in fat tissue and activated to acyl-CoA esters. The inner mitochondrial membrane is impermeable to long-chain fatty acids which are transported into the mitochondria via the carnitine shuttle. Carnitine is transported into cells by a high-affinity carnitine transporter. Within the mitochondria, several chain-length specific enzymes shorten acyl-CoA by two carbon atoms (one acetyl-CoA) in subsequent β-oxidation cycles. Most of the long-chain specific enzymes are associated with the inner mitochondrial membrane (Ogilvie *et al.* 1994; Souri *et al.* 1996; Souri *et al.* 1998; Andresen *et al.* 1999; Fukao *et al.* 2001; Bartlett and Eaton 2004; Goetzman *et al.* 2007; McAndrew *et al.* 2008; Liang and Nishino 2011; Schiff *et al.* 2013; Zhang *et al.* 2014; Miller *et al.* 2015).

Inherited disorders of mitochondrial β-oxidation can present with great variability. In humans, insufficient ketone body production during catabolic states (prolonged fasting, surgery, infection etc.) may cause hypoketotic hypoglycemic coma sometimes accompanied by signs of hepatic failure. Accumulation of toxic long-chain acylcarnitines, particularly in long-chain fatty acid oxidation disorders, may cause lactic acidosis, cardiomyopathy and hepatopathy similar to some mitochondriopathies. Milder deficiency variants of long-chain fatty acid oxidation and the carnitine shuttle may affect skeletal muscle and manifest as chronic weakness, myalgia or rhabdomyolysis (Zschocke and Hoffmann 2011).

In humans, acyl-CoA dehydrogenase very long-chain deficiency (ACADVLD; OMIM 201475) is considered to be the most common inherited disorder of mitochondrial long-chain fatty acid oxidation (Tucci 2017). The acyl-CoA dehydrogenase very long chain (ACADVL; EC 1.3.8.9) is specific for fatty acids with 16 to 20 carbon atoms and is encoded by the nuclear *ACADVL* gene. While all other acyl-CoA dehydrogenase family members form homotetrameric structures with a monomer size of about 45 kD, ACADVL is a homodimer with a monomer size of about 67 kD due to an extended 180 amino acid C-terminal region, which plays a role in maintaining the quaternary structure and mediates the interactions with the inner mitochondrial membrane (Souri *et al.* 1996; Souri *et al.* 1998; Goetzman *et al.* 2007; McAndrew *et al.* 2008; Schiff *et al.* 2013).

Deficiency of ACADVL (ACADVLD) in humans is an autosomal recessive inherited disorder with considerable allelic heterogeneity and more than one hundred pathogenic variants in the human *ACADVL* gene have been reported until now (Zhang *et al.* 2014; Miller *et al.* 2015). The first human patient was identified as early as 1985 before the era of molecular genetics and the identification of the *ACADVL* gene (Hale *et al.* 1985; Izai *et al.* 1992; Uchida *et al.* 1992; Souri *et al.* 1996). The clinical phenotype is quite variable with respect to the severity of clinical signs and age of onset. Human ACADVLD can be subdivided into three major clinical forms (Andresen *et al.* 1999; Miller *et al.* 2015). A severe neonatal onset form presents shortly after birth with hepatic encephalopathy, Reye-like syndrome, and cardiomyopathy leading to a high mortality. The infantile onset form usually presents with recurrent hypoketotic hypoglycemia and liver dysfunction. The adult-onset form, which occurs during adolescence or later, presents mainly with muscle signs which include myalgia, weakness, episodes of rhabdomyolysis, intermittent or permanently elevated plasma creatine kinase levels and myoglobinuria. Symptoms are triggered by stress such as physical exercise, fasting or cold exposure (Ogilvie *et al.* 1994; Souri *et al.* 1998; Saudubray *et al.* 1999; Fukao *et al.* 2001; Wood *et al.* 2001; Goetzman *et al.* 2007; Liang and Nishino 2011; Schiff *et al.* 2013; Tucci *et al.* 2014; Zhang *et al.* 2014; Miller *et al.* 2015). Histopathological examination of patients suffering from ACADVLD demonstrates the accumulation of lipid droplets within muscles fibers. Therefore, the adult-onset form of ACADVLD may be included in the group of lipid storage induced myopathies (Liang and Nishino 2010; Liang and Nishino 2011).

Elevated tetradecenoylcarnitine (C14:1) is used as a biomarker for the disease. In human medicine, newborn screening based on the levels of acylcarnitines and especially tetradecenoylcarnitine (C14:1) in dried blood spots is widely used to diagnose ACADVLD (Wood *et al.* 2001; Zytkovicz *et al.* 2001; Lindner *et al.* 2010; Miller *et al.* 2015).

In dogs, cases of myopathies have been reported with features suggestive of defects in oxidative metabolism (Platt *et al.* 1999; Biegen *et al.* 2015), but to the best of our knowledge, no genetic variant has been identified so far. Recently, breeders noticed several cases of exercise induced muscle pain and weakness in German Hunting Terriers. The aim of the present study was the characterization of this phenotype, which we term exercise induced metabolic myopathy (EIMM) and the identification of the presumed causative genetic variant.

## Materials and Methods

### Ethics statement

The dogs in this study were examined with the consent of their owners. Blood samples were collected with the approval of the *Cantonal Committee for Animal Experiments* (Canton of Bern; permit BE75/16). All animal experiments were done in accordance with local laws and regulations.

### Breed nomenclature

The Federation Cynologique Internationale (FCI) describes the German Hunting Terrier dog as a compact, well-muscled and hardy hunting dog breed (FCI-st. N° 103/26.05.2015). In this paper, all references to the breed correspond to the FCI standards.

### Animal selection for phenotyping

For the phenotype analyses, German Hunting Terriers with signs of exercise induced intolerance were examined. Control animals consisted of German Hunting Terriers without any signs of exercise induced intolerance, which were presented at the Veterinary Clinic for Small Animals Trier, Germany, for routine medical examinations.

### Clinical examinations

Clinical, neurological and laboratory examinations of dogs were performed at the Veterinary Clinic for Small Animals, Trier, Germany, by a specifically trained thesis student (FM) and a Diplomate of the European College for Veterinary Neurology (MK) to characterize the phenotype of a novel disease associated with exercise intolerance in German Hunting Terriers. Cases consisted of nine German Hunting Terriers with a history of exercise induced weakness, myalgia and pigmenturia. Controls included 14 German Hunting Terrier dogs without signs of exercise intolerance. One affected dog died during the study for reasons unrelated to the described novel disease. Therefore, laboratory findings could only be evaluated in eight affected dogs. Clinical signs and their history after different levels of exercise were further evaluated using a questionnaire filled in by the dog owners.

### Laboratory analyses

Blood samples were taken from the vena cephalica and collected in Li-heparin (Li-H) (Micro tube Sarstedt, Germany), sodium fluoride (NaF; BD Vacutainer, Plymouth UK) and ethylenediaminetetraacetic acid (K-EDTA Sarstedt, Germany) tubes. Using plasma from the Li-H tube, chemistry profiles including the measurement of electrolytes, hepatic enzymes and creatine kinase were obtained using the IDEXX Catalyst Dx Chemistry Analyzer (IDEXX Laboratories, Germany). Blood in NaF tubes was used for measuring the lactate level at the Synlab Laboratory, Trier, Germany. Complete blood cell count was performed using the IDEXX ProCyte Dx Hematology Analyzer (IDEXX laboratories, Germany) with full EDTA blood. The concentration of the brain natriuretic peptide (BNP) was determined from EDTA plasma by the diagnostic test Cardiopet (IDEXX Laboratories, Germany) as described (Fox *et al.* 2015). An acylcarnitine profile screening was performed in the Screening-Labor Hannover, Germany. For this test, EDTA blood was placed onto human newborn screening cards and air-dried. Dried blood spots of 3.2 mm diameter were extracted with 200 µl methanol containing the deuterated internal acylcarnitine standards. After evaporation of the extracts acylcarnitines were butylated using 50 µl of 3 N butanolic HCl at 65° for 15 min. After drying the residue was dissolved in methanol/water 80/20, the mobile phase was acetonitrile/water 80/20. The acylcarnitines were analyzed without chromatographic separation in the positive ion mode, using multichannel analyzer scan (MCA). Long-chain hydroxyacyl carnitines were measured in positive MRM mode. The system consisted of a Waters Micro tandem mass spectrometer equipped with electrospray ionization (ESI) source connected with a LC pump and a PAL autosampler. Urine samples were taken by urinary catheterization and then investigated with urine dipsticks (Multistix Siemens Healthcare Diagnostics Inc, Tarrytown, NY, USA). The urinary sediment was examined under a microscope (Motic B Series, Motic Asia, China). All laboratory parameters are summarized in Table 1.

**Table 1:** Summary of different laboratory parameters and diagnostic tests

**Laboratory tubes**	**Examined parameters**	**Technical devices or external laboratory**
Li-heparin	chemistry profile (Glu, Bun, Cr, Bun/Cr, CK, Phos, Ca, TP, Alb, Glob, Alb/Glob, ALT, ALKP, GGT, TBil, Chol, Amy, Lip)*^a^*	IDEXX Catalyst Dx
NaF	lactate	Synlab Laboratory Trier
K-EDTA	hematology	IDEXX ProCyte Dx
K-EDTA	acylcarnitine screening, multichannel analyzer scan (MCA)	Screening-Labor Hannover
K-EDTA	brain natriuretic peptide (BNP)	Cardiopet IDEXX
Urine	urine specific gravity, leukocytes, nitrite, urobilinogen, protein,pH, erythrocytes, ketones, bilirubin, glucose	Multistix Siemens Healthcare

a Glucose (Glu), urea (Bun); creatinine (Cr); creatine kinase (CK); phosphorous (P); calcium (Ca), total protein (TP); albumin (Alb); globulin (Glob); alanine aminotransferease (ALT); alkaline phosphatase (ALKP); gamma-glutamyltransferase (GGT); bilirubin (TBil); cholesterol (Chol); amylase (Amy); lipase (Lip)

### Biopsy procurement and examination

Muscle and nerve biopsies were taken from two cases (JT007 and JT009) under general anesthesia. Biopsies were taken from biceps femoris and tibialis cranialis muscles as well as from fibular nerve. Samples were shipped on wet ice by overnight service to the neuropathology laboratory of the Ludwig-Maximilians-Universität in Munich where they underwent routine processing. Muscle samples were snap-frozen in isopentane cooled in liquid nitrogen. Cryosections were performed and stained with hematoxylin-eosin, periodic acid Schiff, Engel´s modified Gomori stain and Oil Red O. Additional sections underwent enzyme histochemistry for cytochrome oxidase (COX) and nicotinamide adenine dinucleotide tetrazolium reductase (NADH-TR). Muscle fiber typing was performed immunohistochemically (IHC) using murine antibodies directed against skeletal fast myosin (clone MY-32, Sigma-Aldrich Inc., St. Louis), secondary labeling through polymers (IMPRESS, Linaris Inc. Freiburg) and a horseradish peroxidase-diaminobenzidine tetrahydrochloride detection system.

Further samples of each muscle were fixed in 6.5% glutaraldehyde for embedding in epoxy resin. Semithin sections were taken and stained with azure II methylenblue-safranin O for prelocalisation. Selected areas then were cut at 50 nm thickness, mounted on copper grids, contrasted with lead citrate and uranyl acetate for transmission electron microscopy (Zeiss EM 10, Jena).

Nerve samples were fixed in 2.5% glutaraldehyde, contrasted with osmium tetroxide and likewise embedded in epoxy resin. Semithin sections were stained as mentioned above. Further pieces of osmium stained nerves were subjected to nerve fiber teasing for longitudinal fiber examination. Procedures and diagnostic algorithms concisely have been described, elsewhere (Gross *et al.* 2016).

### Animal selection for genotyping

For the genetic analysis we included 120 German Hunting Terriers consisting of 10 cases and 110 controls. The ten cases consisted of the nine dogs that underwent the detailed clinical and laboratory examinations and one additional dog with owner-reported exercise intolerance. Furthermore, samples from 435 dogs of 60 genetically diverse breeds, which had been donated to the Vetsuisse Biobank, were used as controls (Table S1).

### Reference sequences

All analyses were performed using the dog CanFam 3.1 genome assembly as reference sequence. Numbering within the *ACADVL* gene refers to the NCBI accessions XM_546581.5 (mRNA) and XP_546581.3 (protein) for dogs and NM_000018.3 (mRNA) and NP_000009.1 (protein) for humans. The human and dog proteins both consist of 655 amino acids with 92% sequence identity.

### Whole genome resequencing and variant filtering

An Illumina TruSeq PCR-free library with an insert size of 350 bp was prepared from one affected German Hunting Terrier (JT007) and 334 million 2 × 150 bp paired-end reads corresponding to 37x coverage were obtained on an Illumina HiSeq 3000 instrument. The reads were mapped to the dog CanFam3.1 reference genome assembly and aligned using Burrows-Wheeler Aligner version 0.7.5a with default settings (Li and Durbin 2009). The generated SAM file was converted to a BAM file and the reads were sorted using samtools (Li 2011). Picard tools (http://sourceforge.net/projects/picard/) was used to mark PCR duplicates. To perform local realignments and to produce a cleaned BAM file, we used the Genome Analysis Tool Kit (GATK version 3.6.1, 50; McKenna *et al.* 2010). The GATK software was also used for base quality recalibration with canine dbSNP data as training set.

### Variant calling

Putative single nucleotide and small variants were identified in each sample individually using Broad GATK HaplotypeCaller v3.6 in gVCF mode, and subsequently jointly genotyped using Broad GenotypeGVCFs walker (-stand_emit_conf 20.0; -stand_call_conf 30.0); (Van der Auwera *et al.* 2013). Variants were marked but not removed using VariantFilter GATK module, based on the following specifications: SNPs: Quality by Depth: QD < 2.0; Mapping quality: MQ < 40.0; Strand filter: FS > 60.0; MappingQualityRankSum: MQRankSum < -12.5; ReadPosRankSum < -8.0. INDELs: Quality by Depth: QD < 2.0. Strand filter: FS > 200.0. Functional effects and genomic context of the called variants were annotated using SnpEff (Cingolani *et al.* 2012) software together with the NCBI *Canis lupus familiaris* Annotation Release 104. For private variant filtering we used 191 control genomes. These genomes were either publicly available (Bai *et al.* 2015) or produced during other previous projects (Table S2).

### DNA extraction, PCR and Sanger sequencing

Genomic DNA was extracted from EDTA blood samples using the Maxwell RSC Whole Blood DNA Kit in combination with the Maxwell RSC machine (Promega Corporation, Madison, WI, USA). For targeted genotyping of the *ACADVL* c.1728C>A variant we used a Sanger sequencing protocol. Specifically, PCR products were amplified from genomic DNA using the AmpliTaqGold360Mastermix (Thermo Fisher Scientific Corporation, Waltham, MA, USA) and primers 5‘-TCTTTATGCAGACCGTGCAG-3‘ (forward) and 5‘-ACAGGGAAGGTGGTGTTCAG-3‘ (reverse). After treatment with exonuclease I and alkaline phosphatase, we sequenced amplicons on an ABI 3730 DNA Analyzer (Thermo Fisher Scientific Corporation, Waltham, MA, USA). Sanger sequences were analyzed with the Sequencher 5.1 software (Gene Codes Corporation, Ann Arbor, MI, USA).

### Statistical analysis

Continuous data were expressed as median values and ranges. The data for CK, ALT, C14:1 and lactate levels were tested for normality using the Shapiro Wilk test. To investigate if there were significant differences in the laboratory results between cases and controls Wilcoxon Rank Sum tests were used for non-normally distributed data. We repeated these investigations with respect to the three genotype classes (homozygous mutant, heterozygous, homozygous wildtype) of the previously investigated dogs with Kruskal-Wallis tests. For evaluating differences between genotypes, the level of significance was corrected for multiple testing (z ≥ 2.394; *P* < 0.017). As the plasma lactate levels were normally distributed, a two-samples T-test and a one way ANOVA instead of the previously mentioned tests were applied. All the statistical analyses were performed using the NCSS 11 Statistical Software (NCSS, LLC. Kaysville, UT, USA). The level of significance was set to *P* < 0.05.

### Data availability

Figure S1 illustrates the genotypes of the dogs from the extended family for the *ACADVL*:c.1728C>A variant. Table S1 contains genotypes of 435 control dogs from 60 diverse dog breeds for the *ACADVL*:c.1728C>A variant. Table S2 contains the accession numbers of the sequenced case and the 191 control-genomes used for variant filtering. Table S3 lists the detected private variants and their functional annotation in the affected German Hunting Terrier. The sequence data were deposited under study accession PRJEB16012 and sample accession SAMEA104125120 at the European Nucleotide Archive.

## Results

### Clinical examination

Three female and six male affected German Hunting Terrier dogs with an age range from 7 to 42 months were examined (median 32 months). The dogs showed a history of generalized weakness, exercise intolerance and severe diffuse muscle pain. All owners reported, that their dogs could not be used for hunting, as they all collapsed and developed tetraparesis to tetraplegia after 30 to 120 min of exercise. During these episodes, all affected dogs presented a brownish discoloration of the urine.

On physical examination all dogs were alert and responsive. Two dogs (JT007, JT009) were tetraparetic and palpation elicited diffuse muscle pain. Furthermore, dog JT009 had mild muscle atrophy in the fore- and hind limbs. None of the affected dogs had cardiac or pulmonary abnormalities; the neurological examination did not reveal further abnormalities in addition to the previously described tetraparesis in two dogs. Physical and neurological examinations were unremarkable in all examined 14 control German Hunting Terriers.

### Laboratory findings

Complete blood cell counts were available for 22 German Hunting Terriers (8 cases and 14 controls). All hematological values were within the normal reference ranges. In contrast, chemistry profiles revealed several abnormalities in affected dogs. Plasma creatine kinase (CK) activity was significantly higher in affected dogs (145-15,090 IU/l, median 533 IU/l) than in controls (52-219 IU/l, median 71 IU/l; *P* = 0.0007). Similar findings were obtained regarding plasma alanine transaminase (ALT) activities. Cases (44-2,705 IU/l, median 383 IU/l) had significantly higher ALT activity than controls (28-108 IU/l, median 63 IU/l; *P* = 0.002). The plasma lactate levels were only available for 19 dogs (7 cases and 12 controls). Cases (0.2-1.6 mmol/l, median 1.2 mmol/l) were not significantly different from controls (0.1-2.7 mmol/l, median 1.3 mmol/l; *P* = 0.3). All other chemistry parameters were in the respective reference ranges in the examined German Hunting Terriers.

An acylcarnitine profile was available for 22 dogs. Cases revealed a prominent tetradecenoylcarnitine (C14:1) peak with a range from 0.5-2.6 µmol/l and a median of 1.3 µmol/l. In controls, virtually no tetradecenoylcarnitine (C14:1) was detectable (0-0.1 µmol/l, median 0.0 µmol/l; *P* = 0.00004).

All eight cases excreted a dark brownish urine after exercise. In comparison, urine of controls appeared normal. Urinalyis was in the reference range. Brain natriuretic peptide (BNP) levels, marker for myocardiac disease, were within the reference ranges in four affected dogs tested.

### Biopsy findings

**Figure 1:**
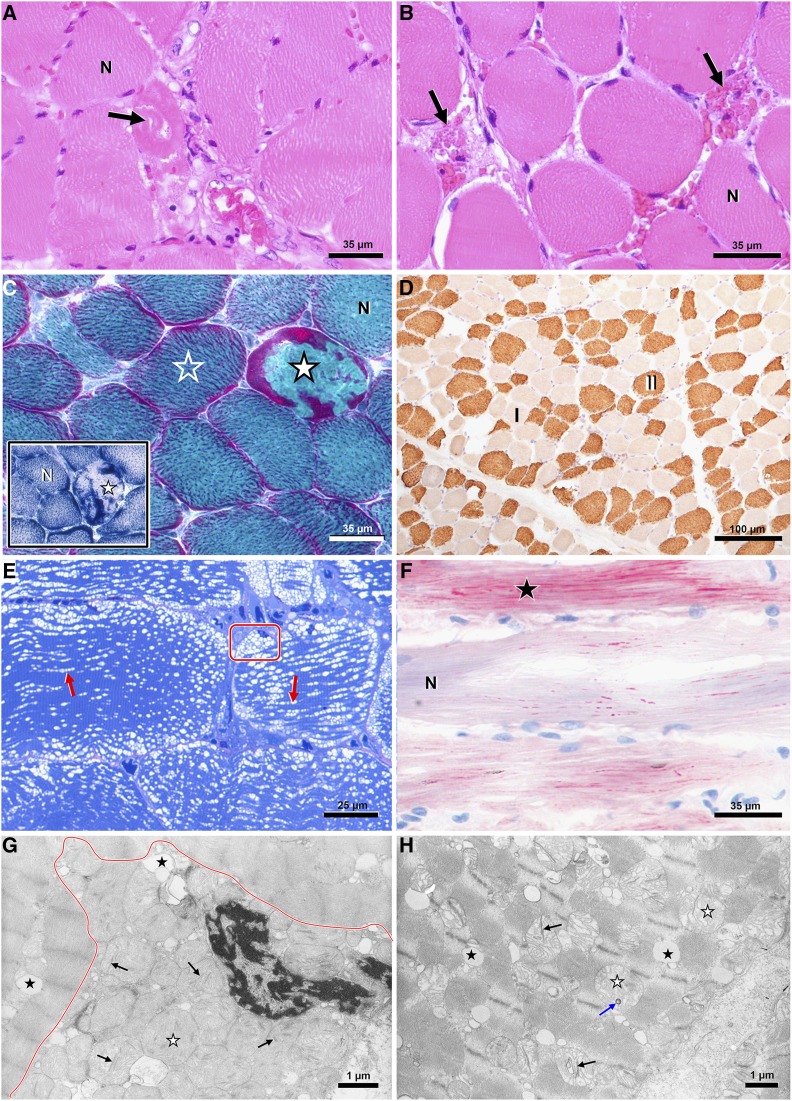
Myopathological characteristics of German Hunting Terriers with exercise induced myopathy (A-H). On light microscopic level, the muscles show widespread individual myofiber necroses (A,B: arrow) at active (A) and resorptive/postresorptive stages (B) surrounded by normal myocytes (A,B: N). (C) Necrotic fibers (filled asterisks) accumulate mitochondria that appear red on Engel´s stain and exhibit high activity of mitochondrial enzyme NADH-TR in histochemical preparations (inlet; black stain). Also non-necrotic fibers (empty asterisk) present with prominent subsarcolemmal and interfibrillar mitochondria if compared to normal fibers (C main/inlet: N). (D) Fiber necroses and atrophy both are predominantly recognized in fatiguable fast-twitch type II fibers (brown), which parallels to relative type I fiber (pale fibers) enrichment. Mitochondrial areas on semithin sections (E) appear vacuolated (frame: subsarcolemmal/perinuclear vacuolation; red arrows: lined interfibrillar vacuoles), which matches with the distribution of lipid droplets in oil red O stained fibers (F; black asterisk), if compared to normal fibers (N). Ultrastructurally, both perinuclear (G) and interfibrillar (H) vacuolated areas contain masses of dysmorphic mitochondria (white asterisks) showing disorganized cristae, trilaminar membranous bodies (black asterisks) and other electron dense inclusions (blue arrow), as well as membrane-bound lipid droplets (black asterisks). Stains: (A,B): hematoxylin-eosin, (C, main): Engel´s modified Gomori, (C, inlet): NADH-TR, (D): anti-fast myosin IHC, (E): azure II methylenblue-safranin O, (F): oil red O, (G,H): lead citrate-uranyl acetate.

Muscle samples harvested from the affected German Hunting Terriers JT007 and JT009 revealed a mild to moderate necrotizing myopathy with enrichment of interfibrillar lipid droplets and mitochondrial abnormalities (Figure 1). Changes were widely spread and more prominent in type 2 fibers. Nerve biopsies from both dogs were unremarkable (not shown).

### Genetic analysis

**Figure 2:**
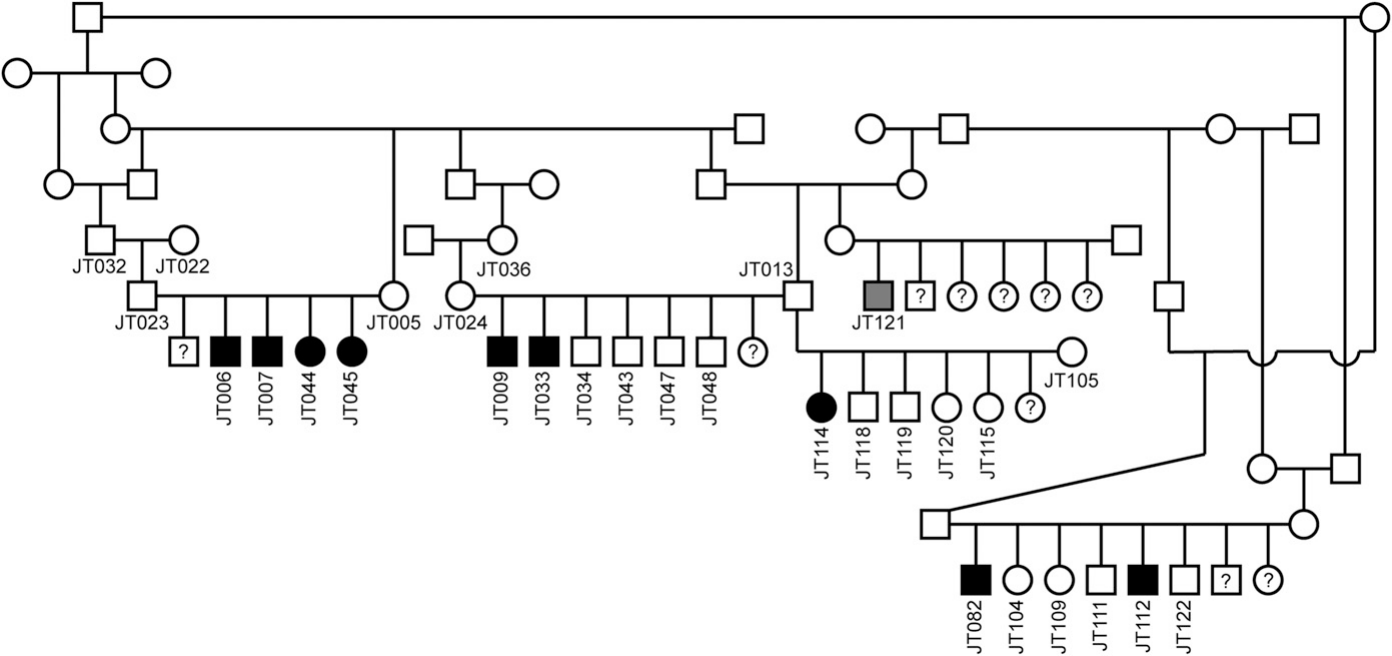
Pedigree of EIMM affected German Hunting Terriers used for this study. Filled symbols represent dogs with signs of myopathy. The gray filled symbol indicates an owner-reported affected dog that was not clinically examined by one of the authors. Numbers indicate dogs from which samples were available. The pedigree was drawn with a limited subset of animals for clarity. Multiple other inbreeding loops are not shown. It was not possible to unambiguously identify the potential founder of the trait.

Given the results of the metabolic and histopathological analysis, the *ACADVL* gene became the primary functional candidate gene for the observed phenotype. In humans, ACADVLD has a strictly autosomal recessive mode of inheritance. In agreement with our hypothesis, pedigree analysis of the extended German Hunting Terrier family strongly suggested an autosomal recessive mode of inheritance. Both male and female dogs were affected. The available cases belonged to five different litters with unaffected parents. The pedigree of these dogs revealed many inbreeding loops (Figure 2).

We performed whole genome sequencing on one affected dog and called single nucleotide and small indel variants with respect to the reference genome of a presumably unaffected Boxer (CanFam 3.1). We searched for private homozygous protein-changing variants by comparing the variants in the case with the genomes of 3 wolves and 188 dogs from various breeds (Table S2). We did not have any specific information regarding a potential myopathy phenotype in the control animals. However, as this is a rare condition, we assumed the control dogs and wolves to be homozygous wildtype at the causative variant.

**Figure 3:**
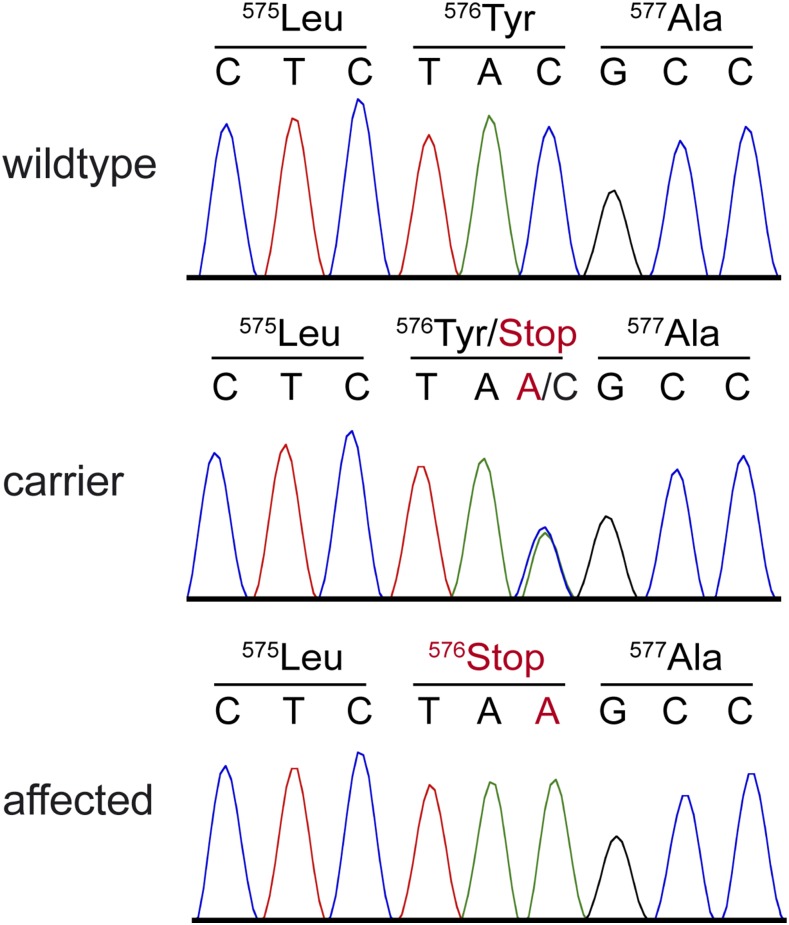
Sanger electropherograms of the *ACADVL*:c.1728C>A variant. A genomic *ACADVL* fragment was amplified by PCR and sequenced with the Sanger method. The figure shows representative data from dogs with the three different genotypes.

The variant calling pipeline detected more than 3 million homozygous variants in the genome of the sequenced case. Of these, 23 were absent from the control genomes and predicted to be protein changing. One of them was located in the *ACADVL* gene, while the other 22 were in genes that we did not consider to be likely functional candidate genes (Table 2; Table S3). The *ACADVL* variant was a nonsense variant, c.1728C>A, predicted to result in a premature stop codon that truncates 80 amino acids from the C-terminus of the ACADVL protein. The formal variant designation on the protein level is p.(Tyr576*). The variant was confirmed by Sanger sequencing (Figure 3).

**Table 2:** Variants detected by whole genome resequencing of an affected German Hunting Terrier. Private variants were exclusively present in the affected dog and had homozygous reference or missing genotype calls in 191 control genomes

**Filtering step**	**Variants***^a^*
Homozygous variants in whole genome	3,129,879
Private homozygous variants in the whole genome	2,555
Private protein changing homozygous variants in the whole genome	23
Private protein changing homozygous variants in the *ACADVL* gene	1

a Only variants which passed the GATK quality filter were counted.

We then genotyped the variant in a cohort of 10 affected and 110 unaffected German Hunting Terriers. This revealed a perfect association of the genotypes with the phenotype (Table 3). All 10 affected dogs carried the variant in homozygous state. Five available parents of affected dogs were heterozygous (obligate carriers; Figure S1). The genotyped cohort was not necessarily representative for the entire population as we preferentially collected close relatives of the affected dogs. In a subset of 90 dogs that were not part of the extended pedigree shown in Figure 2, we observed 15 heterozygous carriers (17%). We also genotyped 435 dogs from genetically diverse breeds (Table S1). None of these dogs carried the *ACADVL*:c.1728C>A variant.

**Table 3:** Association of the *ACADVL*:c.1728C>A genotypes with myopathy

**Genotype**	**C/C**	**C/A**	**A/A**
German Hunting Terriers with signs of myopathy*^a^*	—	—	10
German Hunting Terrier controls*^b^*	81	29	—
Dogs from other breeds	435	—	—

a 9 clinically examined cases included.

b 14 clinically examined controls included.

### Genotype and phenotype correlation

**Figure 4:**
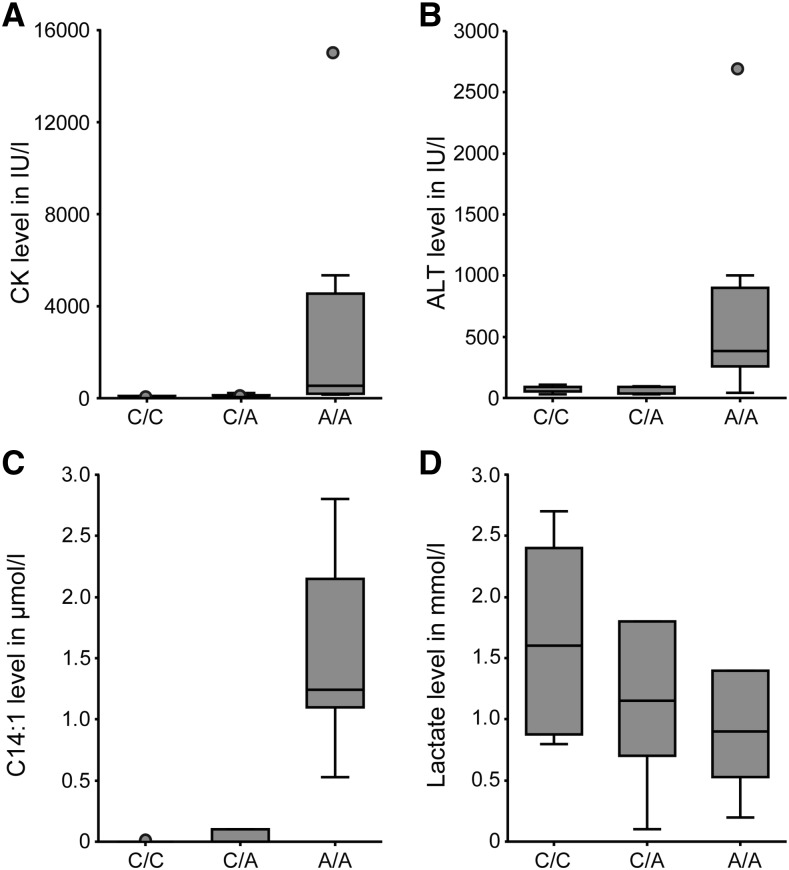
Genotype-phenotype correlation of laboratory parameters. (A) Plasma CK level, (B) plasma ALT level, (C) blood tetracenoylcarnitine (C14:1) level and (D) plasma lactate level. In each of the box plots the distribution of the laboratory parameters with respect to the *ACADVL*:c.1728C>A genotypes are shown. Boxes indicate the range from first to third quartile with medians indicated by solid horizontal lines.

We divided the 22 dogs with laboratory parameters in three groups based on their *ACADVL*:c.1728C>A genotype and re-evaluated the CK, ALT, C14:1 and plasma lactate levels (affected A/A, n = 8; carrier C/A, n = 6; clear C/C, n = 8). The plasma lactate level did not show any significant differences between the three groups. CK, ALT and C14:1 showed significant differences between affected dogs and either carriers or clear dogs. There were no statistically significant differences for CK, ALT and C14:1 between carriers and clear dogs (Table 4; Figure 4).

**Table 4:** Laboratory findings

**Dog ID**	**Sex**	**Age (months)**	**Clinical signs of myopathy**	***ACADVL:* c.1728C>A genotype**	**CK (IU/l)***^a^*	**ALT (IU/l)***^a^*	**C14:1 (µmol/l)***^a^*	**Lactate (mmol/l)***^a^*	**Urine color after strain**	**BNP (pmol/l)***^a^*
JT007	m	32	yes	A/A	15,090	402	1.1	1.4	brown	n.d.
JT009	m	23	yes	A/A	5,354	2,705	1.1	0.8	brown	250
JT033	m	23	yes	A/A	2,174	602	2.4	n.d.	brown	285
JT044	f	32	yes	A/A	145	44	2.6	0.9	brown	356
JT045	f	32	yes	A/A	185	364	1.4	<0.2	brown	408
JT082	m	42	yes	A/A	266	1,000	0.5	1.6	brown	n.d.
JT112	m	42	yes	A/A	799	305	1.1	1.2	brown	n.d.
JT114	m	7	yes	A/A	176	245	1.4	1.4	brown	n.d.
JT005	f	96	no	C/A	52	38	0.0	1.8	yellow	n.d.
JT012	m	15	no	C/A	103	98	0.1	1.2	yellow	n.d.
JT013	m	70	no	C/A	53	62	0.0	1.8	yellow	n.d.
JT018	m	33	no	C/A	64	89	0.1	1.1	yellow	n.d.
JT019	m	56	no	C/A	63	31	0.0	0.9	yellow	n.d.
JT117	m	6	no	C/A	219	36	0.0	<0.1	yellow	n.d.
JT008	f	119	no	C/C	65	96	0.0	n.d.	yellow	n.d.
JT010	m	66	no	C/C	88	73	0.0	n.d.	yellow	n.d.
JT015	f	50	no	C/C	55	28	0.0	2.3	yellow	n.d.
JT016	m	56	no	C/C	74	56	0.0	1.8	yellow	n.d.
JT017	m	55	no	C/C	98	108	0.0	0.9	yellow	n.d.
JT020	m	114	no	C/C	68	82	0.0	1.4	yellow	n.d.
JT021	m	126	no	C/C	80	56	0.0	2.7	yellow	n.d.
JT118	m	24	no	C/C	194	64	0.0	0.8	yellow	n.d.

a Reference values in dog: CK = 48-400 IU/l; ALT = 18-86 IU/l; C14:1 = 0-0.14 µmol/l; lactate = 0.22-1.44 mmol/l; NT- proBNP ≤ 900 pmol/l (Vaden 2009; Osorio and Uribe-Velásquez 2007).

## Discussion

This study comprised clinical, laboratory and histopathological examinations to describe the phenotype of an inherited form of exercise induced metabolic myopathy (EIMM) in German Hunting Terriers. A whole genome resequencing approach revealed the *ACADVL*:c.1728C>A nonsense variant as candidate causative defect for the observed phenotype.

Our claim of causality for the *ACADVL*:c.1728C>A variant is supported by three lines of evidence, perfect co-segregation of the mutant allele in an extended family, perfect genotype-phenotype association in a fairly large cohort of dogs, and the knowledge about the functional impact of *ACADVL* variants in human patients.

We presented clinical data on three female and six male German Hunting Terrier dogs with signs of myopathy with an age range between 7 to 42 months. The observed signs included muscle pain, weakness in all four limbs and exercise induced intolerance after light strain in all nine affected dogs. The diagnosis of myopathy is supported by the rhabdomyolysis found in the muscles biopsies and the elevated plasma CK levels in the affected dogs. CK is an enzyme which is mainly found in the skeletal and cardiac muscle, as well as in the brain and the intestine. An elevated CK level is widely used as marker for muscle cell damage as CK is released into the circulation during cytolysis (Tvarijonaviciute *et al.* 2017). Although no cardiac abnormalities were observed during the clinical examination, plasma BNP levels, a cardiac marker used in humans, dogs, and cats, were measured to assess myocardium integrity in four affected dogs and to exclude or include cardiovascular involvement in the disease process (Andresen *et al.* 1999). All BNP values were within the physiological limits. Rhabdomyolysis present in the biopsies of two affected dogs supports the assumption that the elevated CK values found in myopathic dogs are due to the observed pathological muscle changes. Such findings are also present in the adult-onset form of ACADVLD in humans. Given the absence of cardiac injury, we conclude that EIMM in German Hunting Terriers clinically most closely resembles the adult-onset form of the ACADVLD in humans (Andresen *et al.* 1999; Miller *et al.* 2015). ALT is mostly a specific enzyme of the liver, but can also be found in erythrocytes and skeletal muscle (Nelson and Couto 2014). We observed that the affected dogs had significantly increased plasma ALT values in comparison to the non-myopathic dogs. As other liver function indicators were all within the reference ranges and as the blood count did not reveal any abnormalities, we assume that the liver integrity and the erythrocytes were normal in the affected dogs. Therefore the observed elevated ALT levels most likely reflect muscle damage. This finding also supports the notion that the observed phenotype is similar to the adult-onset form of ACADVLD in humans, as a liver disorder is only found in the early-onset forms of human ACADVLD (Andresen *et al.* 1999; Miller *et al.* 2015).

Using mass spectrometry we discovered a highly elevated level of C14:1 in the acylcarnitine profile in dogs with EIMM. An abnormal elevation of long-chain acylcarnitine such as tetradecenoylcarnitine (C14:1), is characteristic for an inherited disorder of mitochondrial β-oxidation disorders such ACADVLD in humans (Tucci, 2017, Liang and Nishino 2010). The routinely used human cutoff value for this metabolite is 0.25 µmol/l. One published study in dogs suggested a range of 0-0.14 µmol/l for adult healthy dogs (Osorio and Uribe-Velásquez 2007). Our results confirm that C14:1 can be used as laboratory marker for ACADVLD in dogs as there was a significant difference and no overlap between the affected and unaffected animals. EIMM cases had tetradecenoylcarnitine (C14:1) levels ≥ 0.53 µmol/l whereas all controls were ≤ 0.10 µmol/l.

Lactate concentration was previously used to describe other lipid storage myopathies caused by a pyruvate dehydrogenase deficiency (Shelton *et al.* 1998). In our study, the plasma lactacte level was within the reference ranges for all examined dogs. Somewhat unexpectedly, there was no significant difference between cases and controls. Since lactate could not be measured immediately after exercise in the current study, the relatively short half life of lactate could explain these findings (Vaden 2009). Therefore, measurement of lactate levels is not recommended as a marker for the ACADVLD in dogs.

The affected German Hunting Terriers presented a brownish discoloration of the urine during or shortly after a collapsing episode. Such pigmenturia is usually found during episodes of hemolysis due to the presence of hemoglobin or erythrocytes in urine, or during episodes of rhabdomyolysis and myoglobinuria (Shelton 2004). We assume that the observed brownish coloration of the urine was most likely caused by the presence of myoglobin, in agreement with the results found in the muscle biopsies. Episodes of rhabdomyolysis and myoglobinuria have also been described for the adult-onset form of ACADVL deficiency in humans and are significant signs in previously healthy adults who suddenly present with EIMM (Hoffman *et al.* 2006). This represents another potential similarity of the observed phenotype in German Hunting Terriers with ACADVLD in humans (Andresen *et al.* 1999; Miller *et al.* 2015). The histopathological examination of the muscle biopsies revealed lipid droplets within the muscle fibers in addition to the necrotizing myopathy. Similar accumulation of lipid droplets within muscle fibers is found in the adult-onset form in human patients with ACADVLD (Liang and Nishino 2010, Liang and Nishino 2011).

Mitochondrial fatty acid oxidation is assumed to be similar in both humans and dogs and the latter rely heavily on free fatty acid oxidation at all levels of exercise intensity compared to humans (Hill 2010). Endurance dogs, and here we include German Hunting Terriers, primarily use free fatty acid oxidation to provide ATP for exercise (Hill 2010). Therefore, it is not surprising that a reduced or lacking activity of a long-chain fatty acid oxidation enzyme would lead to severe exercise intolerance given the fact that dogs preferentially mobilize long-chain fatty acids during exercise (McClelland *et al.* 1995).

In humans, *ACADVL* variants may lead to autosomal recessive ACADVLD (OMIM #201475). The EIMM in German Hunting Terriers described in this study is also full recessive. Carrier dogs did not show any detectable phenotypic difference in comparison to clear dogs. We were not able to trace the affected animals back to a common founder, suggesting that the *ACADVL*:c.1728C>A variant has been present for several generations in the breed and may be quite widespread. The canine *ACADVL*:c.1728C>A variant is located close to the homologous position of three known human pathogenic variants, p.Arg567Gln (eight cases, Schiff *et al.* 2013), p.Ser583Trp (three cases, Souri *et al.* 1998) and p.Ser583Leu (three cases, Buján *et al.* 2014). Moreover, human *ACADVL* frame-shift variants, comparable to the dog variant, have also been described: p.Leu575Profs*17 and p.Met578Ilefs*15 (Miller *et al.* 2015). Unfortunately, the human patients with these variants were not clearly classified into one of the three forms of ACADVLD (Andresen *et al.* 1999). In humans, it was shown that the C-terminal 180 amino acids of ACADVL play a role for homodimer assembly and are important for the interaction between the protein and the inner membrane of the mitochondrion (Souri *et al.* 1998).

The clinical phenotype in the affected German Hunting Terriers was comparatively mild and most closely resembled the adult form of ACADVLD in humans. Further studies will be necessary to clarify whether affected German Hunting Terriers retain some residual ACADVL enzyme function. In a scenario with complete loss of ACADVL function, the relatively mild phenotype in dogs would suggest some physiological differences between humans and dogs that allow the dogs to better compensate for the lack of ACADVL function. Alternatively, as the dog variant is located near the end of the *ACADVL* gene, in the antepenultimate exon, it seems conceivable that a fraction of the mutant transcripts might escape nonsense mediated decay and that the truncated protein is actually expressed. The truncated protein is predicted to have an intact catalytical domain, which might give rise to residual enzyme function, even if homodimer formation and/or the interaction with the inner mitochondrial membrane are compromised. Residual ACADVL enzyme function in homozygous mutant dogs might also very well explain the relatively mild canine clinical phenotype. As mentioned above, further studies including protein function studies are required to answer these open questions.

In conclusion we discovered a novel hereditary disease and provide a first description of the EIMM phenotype in German Hunting Terriers. Furthermore, we identified a nonsense variant in the *ACADVL* gene as most likely underlying genetic defect. Our findings provide a large animal model for a known human disease and will enable genetic testing to avoid the unintentional breeding of affected offspring.

## Supplementary Material

Supplemental Material is available online at www.g3journal.org/lookup/suppl/doi:10.1534/g3.118.200084/-/DC1.
