# Supplementary material for: A Nonsense Variant in the ACADVL Gene in German Hunting Terriers with Exercise Induced Metabolic Myopathy
Source: G3 (Bethesda). 2018 Feb 28;8(5):1545–54. doi: 10.1534/g3.118.200084 (PMC5940147; doi:10.1534/g3.118.200084)
Supplement: Supplementary file 1 [file 1545FigureS1.pdf]

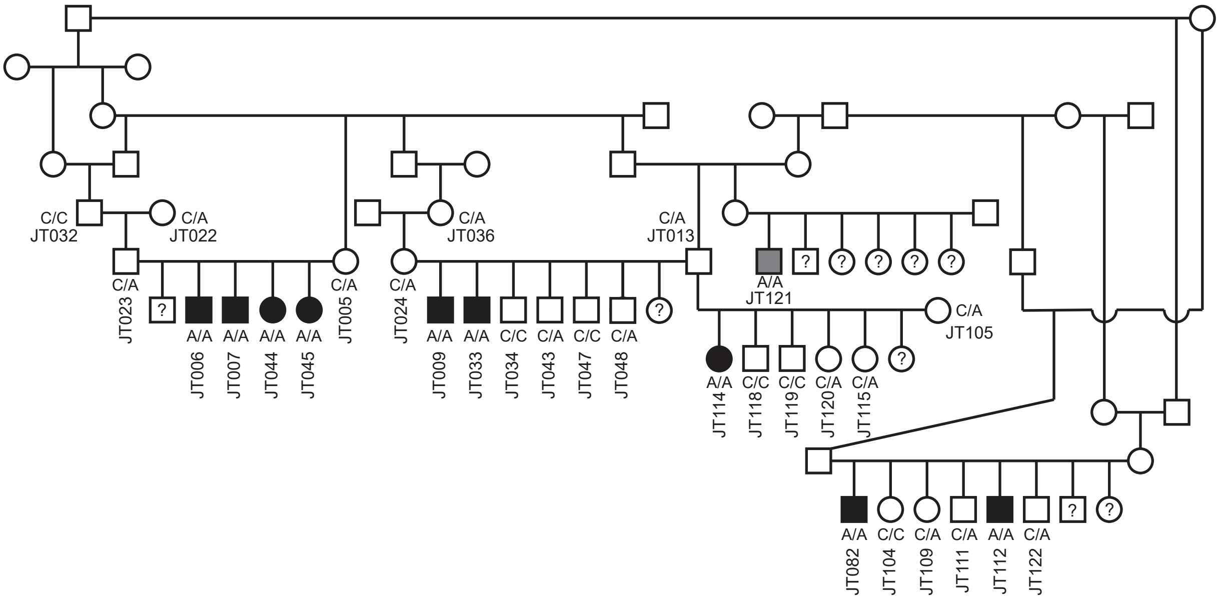

Figure S1. Pedigree of EIMM affected German Hunting Terriers with indicated genotypes at the *ACADVL*:c.1728C>A variant. Note that all affected dogs were homozygous for the A/A genotype. All genotyped parents of affected dogs (= obligate carriers) were heterozygous C/A.
